# Intraosseous arterial alteration of maxilla influencing implant-related surgeries

**DOI:** 10.1007/s00784-023-05141-9

**Published:** 2023-07-17

**Authors:** Arvin Shahbazi, Anton Sculean, Gábor Baksa, Sebastian Gschwindt, Bálint Molnár, János Vág, Sándor Bogdán

**Affiliations:** 1 Department of Anatomy, Histology and Embryology (Oral Morphology Group), Semmelweis University, Budapest, Hungary; 2 Department of Periodontology, Semmelweis University, Budapest, Hungary; 3 Department of Restorative Dentistry and Endodontics, Semmelweis University, Budapest, Hungary; 4 Department of Periodontology, School of Dental Medicine, University of Bern, Bern, Switzerland; 5 Department of Oro-Maxillofacial Surgery and Stomatology, Semmelweis University, Budapest, Hungary

**Keywords:** Implants, Flaps, Wound healing, Blood vessels, Complications

## Abstract

**Objectives:**

To investigate the intraosseous arterial pathways and anastomoses in the alveolar aspects of the maxilla in order to better understand the arterial scattering pattern.

**Materials and methods:**

Eleven cadavers were selected for macroscopic intraosseous arterial analyses by corrosion casting. The red-colored acrylic resin was injected into the external carotid arteries. The specimens were kept in an enzymatic solution at 36 °C for about 60 days, depending on the process progression. After removal of the soft tissues and drying, the bone was macerated by potassium hydroxide to analyze the course and the mean diameters of the intraosseous anastomoses.

**Results:**

Vertico-oblique and horizontal intraosseous arteries and anastomoses between the greater palatine-, posterior superior alveolar-, and infraorbital arteries were detected. The vertico-oblique anastomoses were found on the anterolateral wall of the maxilla and the alveolar crest with a mean diameter of 0.46 mm; nevertheless, the horizontal (transalveolar) anastomoses were identified in the interdental septum/alveolar crest with the mean diameter of 0.41 mm. From the horizontal anastomoses, small intraseptal branches supplied the territory of the alveolar socket in various directions.

**Conclusions:**

The localization of intraosseous arterial anastomoses is critical in implant-related surgeries, predominantly to maintain proper circulation.

**Clinical relevance:**

Based on vertico-oblique and transalveolar anastomoses, simultaneous buccal- and palatal flap elevation (particularly on the palatal side) should be avoided to minimize patient morbidity and intra- or postoperative complications. Moreover, preserving transverse loops in the interdental septum is essential during implant surgeries, which can significantly influence collateral periosteal and osteal circulation to prevent ischemia.

## Introduction

Oral rehabilitation with dental implants is nowadays a well-established treatment modality for replacing missing teeth to restore chewing function and esthetics [1]. Despite the relatively high predictability of implant therapy and high costs, patient perceptions of success and patient-reported outcome measures have become increasingly significant in implant dentistry.

In perioimplant-related surgeries, the morphological and vascularization factors in the maxilla convey an intricate pattern that can result in challenging circumstances [2–4]. Through a better understanding of the topography of the mucosal and periosteal branches of critical arteries, such as the posterior superior alveolar artery (PSAA), greater palatine artery (GPA), and infraorbital artery (IOA) with their anastomoses [5–7], clinicians can avoid or minimize complications related to intense intra- and postoperative hemorrhage, compromised wound healing, and flap necrosis. Previous investigations regarding subepithelial connective tissue grafting revealed that flap design is significantly affected by the morphology of the palatal vault and the topography of the arterial pathway of GPA with critical anastomoses [7–10].

Regarding the intraosseous vascularization patterns in the bony alveolus and implant area, the literature indicates various endoscopic investigations assisting in comprehending vascular canals [11, 12]. Particularly, Engelke et al. [11] demonstrated in vivo patterns of vascular canals (50 microns) and hemorrhage by utilizing microscopic bone analysis and support immersion alveoloscopy [11]. Furthermore, via morphometric investigation, they detected deficient vascular canals and unmineralized bone in the implant zones corresponding to the extraction sockets [11]. During or prior to implant placement, ridge augmentation is frequently inevitable. According to Tan et al. [13], after tooth loss, the vertical dimension of the bone can decrease by 11–22% (0.84 ± 0.62 mm mesially and 0.80 ± 0.71 mm distally) after six months, and the horizontal dimension by 29–63% (3.79 ± 0.23 mm), respectively [13]. Based on the fact of alveolar resorption, the allocation pattern of the intraosseous and periosteal arteries might be altered. Horizontal ridge augmentation is typical in the esthetic zone and premolar area, sometimes along with sinus floor elevation in the posterior region [14]. However, vertical ridge augmentation is predominantly performed in the esthetic zone [15, 16]. In the maxilla, donor sites for autogenous bone harvesting mainly include the maxillary tuberosity and the anterior nasal spine, which contain dense arterial supply and can be impaired during harvesting and alveolar reconstruction. Furthermore, hemorrhage can occur during autogenous bone harvesting from the maxillary tuberosity or during implant placement, especially in the vicinity of the incisive canal, following damage to the nasopalatine artery (NPA) [17].

Several studies have investigated the macroscopic vascularization of the PSAA, IOA, and GPA [5–9]. Regardless, the explicit branching and anastomoses pattern of intraosseous and periosteal arteries remains demanding.

Thus, the present study aimed to analyze the intraosseous vascularization and anastomoses of the arterial network in the alveolar aspects of the anterolateral wall of the maxilla and hard palate, as well as the alveolar crest (interdental septum) by the corrosion casting technique to provide indications for improving the safety of implant-related surgery.

## Materials and methods

Eleven maxillae (7 males, 4 females; 50–85 years of age) were selected for the macroscopic intraosseous arterial investigation (2 dentate, 2 partially edentulous in premolar and molar area, and 7 edentulous). Fresh bodies were provided for corrosion casting analysis. The specimens were selected based on the proper conditions of the vessels without known vascular-related comorbidities. The bodies were donated for science and research to the Department of Anatomy, Histology and Embryology, Semmelweis University, Budapest, Hungary, according to Hungarian approval laws of anatomical donation (approval number: 110/2020.(VII.07.)). In the first step, the external carotid arteries (ECAs) were irrigated by saline solution. Then, acrylic resin (ACRIFIX 190 (2 R 0190), Evonik Industries AG., Germany) was mixed with red Akemi Akepox coloring paste (AKEMI GmbH., Nurnberg, Germany). The mixture was introduced into the ECAs. Subsequently, the head specimens were kept at room temperature for 24 h for complete resin polymerization. In order to dissolve the soft tissues around the injected vessels, the specimens were kept in the enzymatic solution (Somat gold 12 actions (Henkel AG., Germany)) at 36 °C for about 60 days, depending on the process progression. Every 15–20 days, the solution was replaced with great care to avoid a fracture of the intermediate corrosion cast. After all soft tissues were dissolved, the specimens were carefully washed and left in cold water for 3 days to eliminate the remaining chemicals. After drying, the bony areas were macerated by 2–4% KOH to analyze the intraosseous branches. The diameters of vertico-oblique- and horizontal intraosseous arterial anastomoses were measured at the level of the alveolar crest/interdental septum by Topex caliper (31C616 Vernier Caliper 200 mm, Warsaw, Poland), and the mean diameters of anastomoses were compared.

## Results

**Fig. 1:**
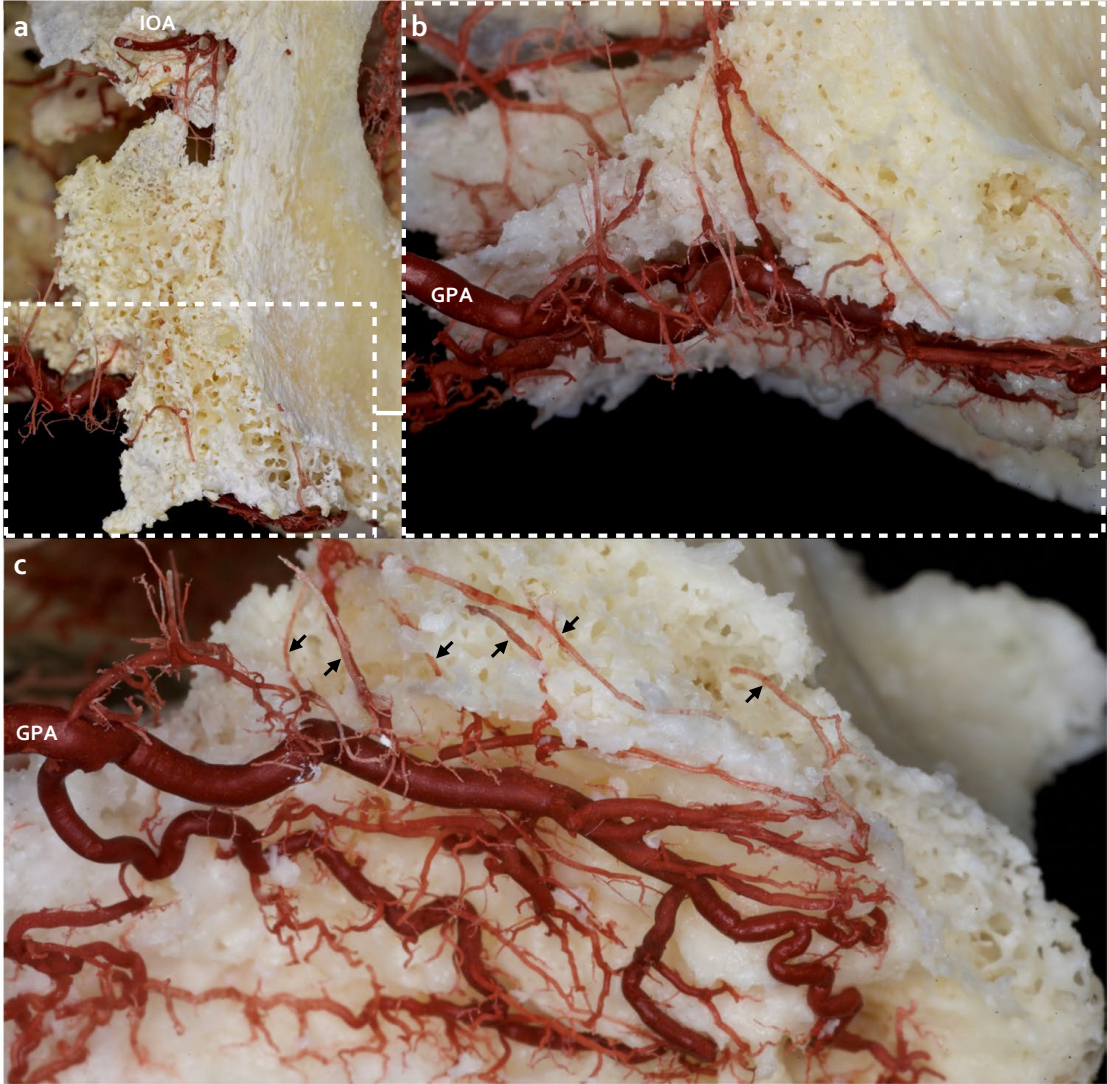
Vertico-oblique intraosseous anastomoses (vertico-oblique loop). (**a**) Anterolateral view of the maxilla, vertico-oblique intraosseous branches of the infraorbital artery (IOA), and the greater palatine artery (GPA), concealed in the anterolateral surface of the maxilla. (**b**) Anterolateral view of the maxilla with a magnified view. After removing the hard tissue through the cautious maceration, vertico-oblique loop between IOA and GPA in the alveolar crest and socket initiated the arterial network. (**c**) Inferior view from the side of the hard palate; the anastomoses are marked with arrows

**Fig. 2:**
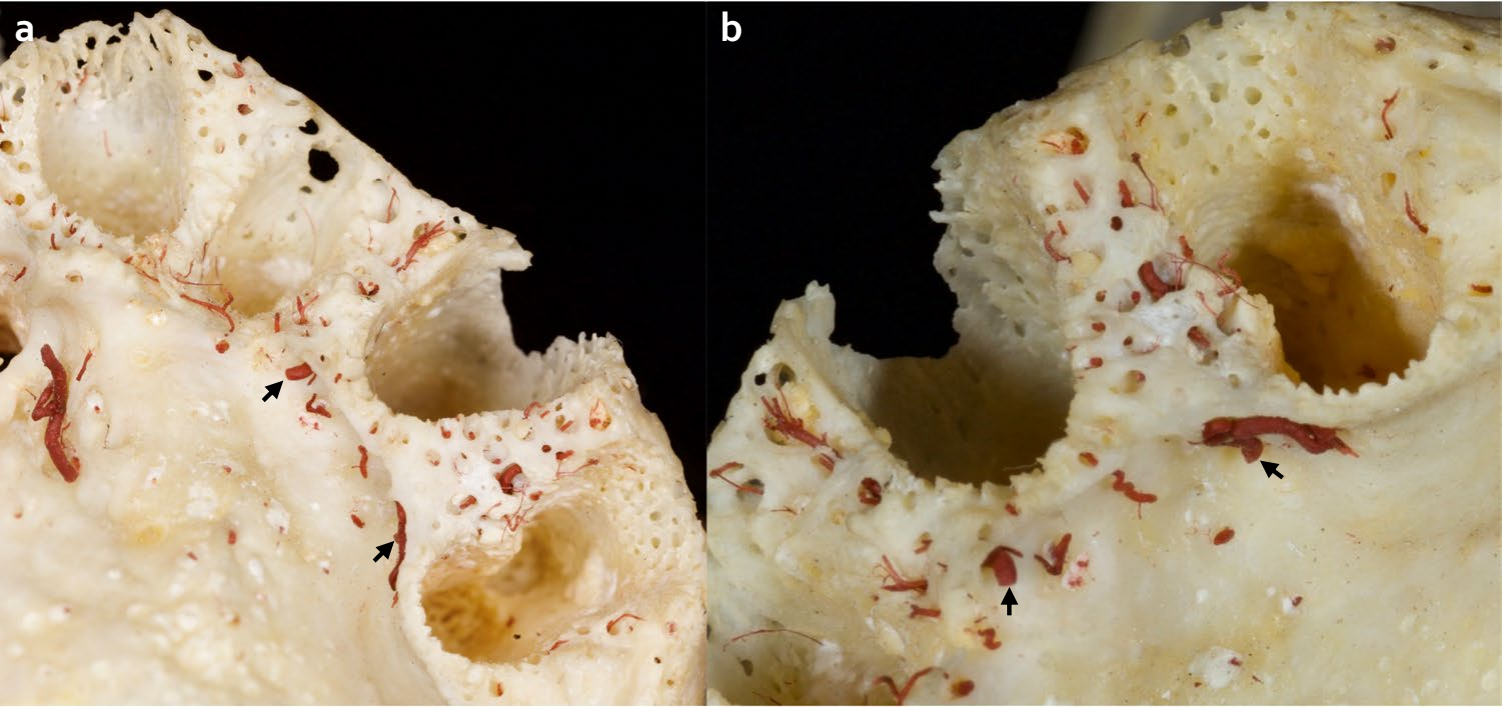
Horizontal intraosseous anastomoses (transverse loop). (**a**) Overview of left front teeth after extraction, transalveolar anastomoses in the interdental septum marked with arrows. (**b**) Marked transverse loop; the intraosseous artery at the palatal aspect exhibits a larger dimension than the vestibular side, and small intraseptal branches are observed

In all investigated specimens, the following arterial connections were discovered: vertico-oblique intraosseous anastomoses (vertico-oblique loop) between branches of PSAA/IOA with the GPA were discovered following maceration of the anterolateral wall of the maxilla and the alveolar crest (Fig. 1). In the interdental septum/alveolar crest, horizontal bony perforating anastomoses (transverse loop) between the GPA branches and the PSAA/IOA branches were identified (Fig. 2). The vertico-oblique anastomoses presented as more prominent in diameter than the horizontal anastomoses (Table 1). Furthermore, the size of the horizontal intraosseous perforating arteries was thicker on the palatal side than the buccal arteries, suggesting that the direction of the blood flow might be from the palatal side toward the buccal/labial side (Fig. 2). From the transverse loop, small intraseptal branches created a network at the territory of the alveolar socket and in a coronal direction they were detected in the alveolar crest (Fig. 2).

**Table 1:** Anatomical locations and mean diameter of the intraosseous arterial anastomoses

Intraosseous anastomoses	Anatomical location	Mean diameter of the arterial anastomoses (level: alveolar crest/Interdental septum)
Vertico-oblique intraosseous anastomoses	Anterolateral wall of the maxilla Alveolar crest	0.46 mm
Horizontal intraosseous anastomoses	Interdental septum Alveolar crest	0.41 mm

## Discussion

Collateral vascularization should be preserved during implant-related surgeries to maintain proper circulation [18, 19]. Inadequate blood supply or damaged bilateral blood flow in the surgical area might increase the risk of ischemia and necrosis in soft and hard tissues [20].

In this study, the corrosion casting method permitted to observe that (i) vertico-oblique intraosseous branches of PSAA/IOA supplying the alveolar aspects of the anterolateral wall of the maxilla formed anastomoses with the intraosseous branches of GPA (vertico-oblique loop); (ii) horizontal perforating transalveolar anastomoses between the branches of GPA with PSAA/IOA (transverse loop) in the alveolar crest/interdental septum presented small intraseptal branches. These intraseptal branches supplied the territory of the alveolar socket and moved toward the alveolar crest, periosteum, and the gingival papilla. Based on our investigations, intraseptal branches assembled anastomoses with the supraperiosteal arteries and the vessels of the periodontal ligament. Furthermore, this network might impact supplying the circulation of the gingiva and can substantially influence the outcomes and complication rate of implant-related surgeries in the maxilla. A previous study from our group regarding maxillary vestibule arterial supply emphasized the bony penetrating branches in connection with transverse mucoperiosteal anastomoses on the buccal aspect in the premolar/molar region which were perfusing the buccal wall and interalveolar septum [21]. The current investigation mainly demonstrates the course of perforating intraosseous arteries and analyzes anastomoses in the alveolar aspects of the maxilla. Conceivably the intraosseous branches may connect with the transverse periosteomucosal network; so in implant-related surgeries, if these anastomoses are mostly preserved, they can play a critical role in the proper perfusion around implant territory. Furthermore, the intraosseous arterial pattern of the NPA branches in the alveolar crest and hard palate should be investigated individually, which could be critical for circulation in the esthetic zone in implant- and maxillofacial related surgeries.

The mean arterial diameter of vertico-oblique anastomoses was slightly more prominent than horizontal anastomoses. However, several physiological factors, such as bone resorption and vascular pressure changes, might influence the dimensions of the anastomoses. Due to the limited numbers of the maxillae, detailed statistical analyses were not assembled. Based on our determinations, horizontal perforating arteries drive from the palate toward the buccal cortex and create an arterial network around the alveolar socket. Preserving transverse loop vascularization in the interdental septum is essential during immediate implant surgeries. The findings also suggest that palatal flap elevation should be avoided or minimized in order to decrease patient morbidity and complications.

During ridge augmentation, the interruption of palatal transalveolar arteries should be avoided in order to facilitate the optimal incorporation of the bone grafts/bone substitutes. Similarly, it seems to be desirable that the implant dimension should be less than the alveolar socket from the mesiodistal and buccopalatal aspects, to minimize the pressure on the bony walls and transalveolar anastomoses thus preventing insufficient circulation and resorption of bony tissue. Additionally, the distance between implants should be at least 2–3 mm to have sufficient interimplant vascularization, especially in the esthetic zone [22, 23]. Nevertheless, small palatal perforating vessels have a poor prognosis and might rupture after excessive flap elevation and mobilization.

Ultimately, in sinus floor elevation, especially in the lateral window technique, it is inevitable to elevate a buccal mucoperiosteal flap and the Schneiderian membrane [24]. Consequently, the blood flow of the alveolar process is bidirectionally compromised due to the interruption of vertico-oblique branches by soft/hard tissue manipulation. The present analysis indicates vertico-oblique loops and transverse loops between GPA and PSAA/IOA are critical anastomoses during lateral sinus floor elevation. Damaging these intraosseous anastomoses might result in disturbed graft blood supply and osteogenesis. The graft vascularization can be ensured by elevating the Schneiderian membrane and reaching the mesial wall; four intact bony walls can provide excellent blood supply utilizing the arterial loops.

## Conclusion

A thorough knowledge of the intraosseous arterial supply is essential to prevent intra- and postoperative complications in implant-related surgeries. Our study highlighted that the intraosseous vascularization of the alveolar aspects of the maxilla strongly relies on the vertico-oblique and transalveolar intraosseous spongious branches of GPA and PSAA/IOA, forming the vertico-oblique and the transverse loop anastomoses. Therefore, simultaneous buccal- and palatal flap elevation should be avoided to minimize patient morbidity and reduce complications during maxillary osseous surgeries.
